# Image-guided facet joint injection

**DOI:** 10.2349/biij.7.1.e4

**Published:** 2011-01-01

**Authors:** WCG Peh

**Affiliations:** National University of Singapore, Singapore

**Keywords:** Facet syndrome, intra-articular facet injection, imaging-guided injections, interventional spinal procedures, low back pain, spinal pain

## Abstract

Chronic spine pain poses a peculiar diagnostic and therapeutic challenge due to multiple pain sources, overlapping clinical features and nonspecific radiological findings. Facet joint injection is an interventional pain management tool for facet-related spinal pain that can be effectively administered by a radiologist. This technique is the gold standard for identifying facet joints as the source of spinal pain. The major indications for facet injections include strong clinical suspicion of the facet syndrome, focal tenderness over the facet joints, low back pain with normal radiological findings, post-laminectomy syndrome with no evidence of arachnoiditis or recurrent disc disease, and persistent low back pain after spinal fusion. The contraindications are more ancillary, with none being absolute. Like any synovial joint degeneration, inflammation and injury can lead to pain on motion, initiating a vicious cycle of physical deconditioning, irritation of facet innervations and muscle spasm. Image-guided injection of local anesthetic and steroid into or around the facet joint aims to break this vicious cycle and thereby provide pain relief. This outpatient procedure has high diagnostic accuracy, safety and reproducibility but the therapeutic outcome is variable.

## INTRODUCTION

The spine is the most common source of chronic pain [1] and the second most common reason for a patient to consult a physician [2]. About two-thirds of the population suffers from back pain at some point of time during their life span [2] and this symptom incapacitates 20% of them for long periods (>4 weeks) [3]. Chronic back pain entails suffering and disability of considerable ergonomic significance, since the majority afflicted by this malaise belongs to the age group of 30–50 years [4]; this implies loss of precious man-hours. The incidence of chronic spine pain is at least 5% annually [5–7], with the average prevalence in adults being 15% [8, 9]. Notwithstanding the fact that duration of pain and its chronicity are controversial topics with poor universal consensus on the definition, pain that continues for more than 7–12 weeks despite conservative management is generally accepted as chronic [4]. Traditionally, it has been believed that most episodes of spinal pain will be short-lived and that 90% of patients recover in about six weeks with or without treatment, and hence it is best managed conservatively, with rest, physiotherapy and analgesics/muscle relaxants [5, 6, 10, 11]. However, several studies have dispelled this belief and shown that chronicity or recurrence of low back pain ranges from 28% to 75% [10, 12–17]. This statistical detail warrants the allopaths to have a keener look at remedies for chronic spinal pain.

In most instances, spinal symptoms are due to a benign non-emergent condition involving some degree of spinal degeneration. Unfortunately in most cases, imaging abnormalities do not correlate with clinical symptoms [18–20]. It is not unusual to encounter an asymptomatic individual with striking abnormalities at imaging [19, 20] and patients with incapacitating symptoms but normal CT and MR findings [18]. The association between symptoms and imaging findings is weak as imaging studies are only able to identify a cause for spinal pain in just 15% of patients with chronic back pain [2, 21]. Hence many patients do not receive a specific diagnosis and continue to suffer pain. In such patients, spinal injections allow a functional assessment of the anatomical structures that could potentially be the culprit. Diagnostic spinal injections can identify the source of pain, and may be followed by therapeutic injections with a mixture of a long-acting local anaesthetic and depot steroid preparations, if relevant.

For an anatomical structure to be deemed as a potential cause of pain, it must fulfil four criteria: a) it must have a nerve supply; b) it should be capable of causing pain similar to that seen clinically in normal volunteers; c) it must be susceptible to painful diseases or injuries; and d) using diagnostic techniques of known reliability and validity, the structure must be demonstrated as a source of pain in patients [22]. Going by these postulates, structures that may cause spinal pain include the vertebral body, inter-vertebral discs, the spinal cord, nerve roots, facet joints, ligaments, muscles, and sacroiliac or atlanto-axial and atlanto-occipital joints. Among these, a radiologist often addresses the following 3 major conditions: (a) disc disease, (b) facet syndrome, and (c) vertebral body disease [23]. Facet joints were proven to be the culprit in 15% to 45% of patients with low back pain [24–29], 54–67% of patients with neck pain [30–32], and 48% of patients with thoracic pain [8]. In short, facet joint is a common cause for chronic pain and this review aims to emphasise on various aspects of imaging-guided intra-articular injection into the facet joint as a diagnostic and therapeutic tool.

## HISTORICAL BACKGROUND

An imaging-guided technique with fluoroscopy or computed tomography (CT) increases the precision of spinal injection procedures and confirms the needle placement. Since they give better results and reduce complications, they are now preferred over blind injections. Goldthwait [33] in 1911 first described lumbar facet joints as a source of back pain, and this was further endorsed by the writings of Putti [34], an Italian surgeon, in 1927. Subsequently, in 1933, Ghormley [35] coined the term facet syndrome and defined it as a lumbosacral pain (with or without sciatic pain) that suddenly occurs after a twisting or rotary strain to the lumbosacral spine. In 1941, Badgley [36] suggested that facet joints could be a primary source of pain separate from the nerve compression component and demonstrated that facet joint pathology could cause symptoms, including radiation of pain into the lower extremities. However, it was only in 1963 that Hirsch et al. [37] injected hypertonic saline into facet joints and experimentally reproduced low back pain along the sacroiliac and gluteal areas with radiation to the greater trochanter. In 1976, Mooney and Robertson [38], followed by McCall et al. (39) in 1979, used fluoroscopic guidance for facet joint injection with steroids and local anaesthetics.

## FACET JOINT ANATOMY [22, 37, 40–52]

**Figure 1:**
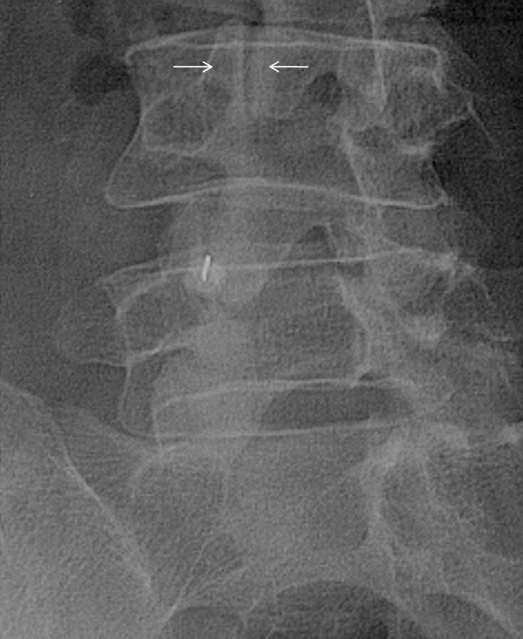
Diagrammatic representation of facet joint anatomy.

The facet joints are paired diarthrodial articulations located between the posterior elements of the adjacent vertebrae; also known as zygo-apophyseal joints. They are formed by the articulation of the inferior articular processes of one vertebra with the superior articular processes of the vertebra below (Figure 1) [40]. They are typical synovial joints with the facets covered by articular cartilage, bridging synovial membrane, tough fibrous capsule and intervening layer of loose areolar tissue. On the ventral aspect, the capsule is deficient and the joint is in contact with the ligamentum flavum. The joint capsule is redundant along the superior and inferior aspects, forming the respective recesses which are filled with small synovial villi or fat pads approximately the size of rice grains [41]. Adipose tissue contained within the superior recess is continuous with that around the spinal nerve [37]. The facet joints are asymmetric in 30 % of the population [43].

**Figure 2:**
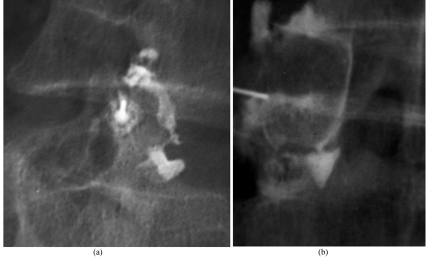
This line diagram demonstrates the dual nerve supply of the facets. Note the dorsal branches (shown by arrows) supplying the facet joint at the level of its exit and the subsequent lower one.

The facet joints are richly innervated by the nerve fibres from the medial branch of the dorsal ramus of spinal nerves. Each facet has a dual nerve supply (Figure 2), from the dorsal ramus at the same level as well as from the level above. Each spinal nerve root innervates two facets; it supplies the facet joint at the level it exits, as well as the subsequent lower facet. The exceptions to this dual nerve supply are: singular nerve supply to the atlanto-occipital joint, atlanto-axial joint and C2/3 facet joint, which are innervated by C1, C2 and C3 nerves respectively [51,52]. Histological studies have shown that capsules of the facet joints are richly innervated with free nerve endings [37, 22]. This means that they are endowed with the appropriate sensory apparatus to transmit proprioceptive and nociceptive information.

**Figure 3:**
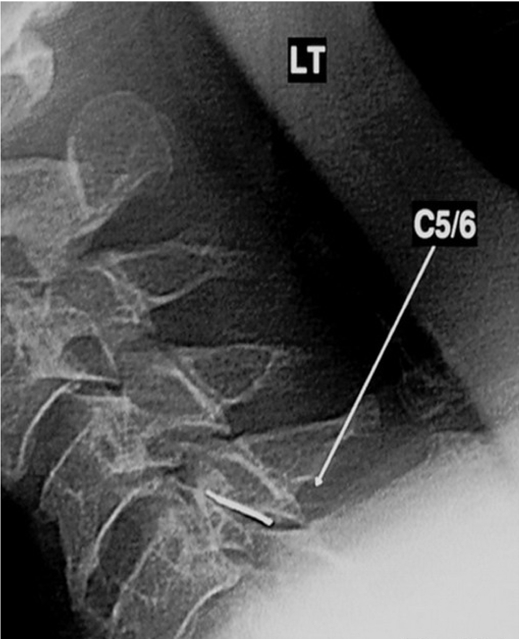
This ceramic model of the spine shows the gradual change in the orientation of the facets. Note the obliquity at which the L3/L4 joint space is seen in profile while the rest of the facet joints are out of profile. It is evident that, starting from a prone position, the upper lumbar facet joints (near sagittal) will be seen in profile at lesser angles of rotation than the lower lumbar facets (near coronal).

The facet joints are anatomically designed to restrain excessive mobility and distribute axial loading over a broad area. The variation in their shapes and their orientation prevents forward displacement and rotatory dislocation of the intervertebral joint. The articular processes do provide a sliding surface for some movements, with roughly 5 to 7 mm of motion possible along the plane of the joint [43]. The cervical facet joints are typically oriented in an oblique coronal plane, angled superior to inferior in a posterior direction. The thoracic facet joints are nearly vertical and coronal in orientation, rotating towards the sagittal plane near the thoracolumbar junction. The superior lumbar facet joints are oriented in a nearly sagittal plane, and the plane of orientation rotates outward towards the coronal plane with descent in the lumbar spine so that the joints are in a sagittal-coronal oblique plane at the lumbosacral junction (Figure 3). Familiarity with the orientation of the joints is important in selecting an appropriate approach for advancing the needle during facet joint injections.

## PATHOPHYSIOLOGY OF THE FACET SYNDROME

Pain of facet joint origin is attributed to a variety of causes, including: segmental instability, synovitis, synovial entrapment, trauma, meniscoid impingement, chondromalacia and osteoarthritis [18, 53–55]. The earlier described synovial villi within the facet joints may become inflamed or trapped between the articular processes, thereby producing pain [40]. Distension and inflammation of the synovial capsule, with resultant stimulation of the nociceptive nerve endings [56] and expanded synovial recesses compressing the nerve roots in the spinal canal and neural foramina, thereby causing radiculopathy in patients with facet syndrome [56, 57], are among the other theories on the subject. Irrespective of the mechanism, degeneration, inflammation and injury of facet joints can lead to pain upon joint motion. This pain leads to restriction of motion, and eventual physical deconditioning. Irritation of the facet joint innervations can also result in muscle spasm. Pain innervations are also present in other local soft tissue structures adjacent to the joint including the multifidus, the local spinal nerves, and the dura and epidural space. Joint inflammation may cause localised hyperemia and venous stasis, thus affecting other local tissues [44]. Thus, a self-perpetuating painful mechanism is set into motion. Certain studies have proposed disc degeneration to initiate degenerative changes in the facet joints by altering the bio-mechanics of the entire motion segment [58, 59]. Numerous other causes like rheumatoid arthritis, ankylosing spondylitis and capsular tears, have also been described as causes of facet joint pain [22]. Radiographic changes of osteoarthritis are equally common in patients with and without low back pain, and degenerative joints seen on CT are not always painful, although some researchers claim that severely degenerated joints are more likely to be symptomatic (19, 22, 60, 61).

## INDICATIONS FOR FACET INJECTION

Imaging findings are of little help in diagnosing the facet syndrome. It is usually a diagnosis of exclusion that is arrived at, after excluding mimics like nerve entrapment syndrome, discogenic pain, spinal stenosis and osseous abnormalities. In patients with facet joint syndrome, distension of the joint with saline or contrast will reproduce the pain, and injection of a local anaesthetic agent will relieve the pain [62, 63]. This response pattern is the current gold standard for diagnosing the facet syndrome [38, 60, 61, 64–66]. Provocation of pain from a joint injection is an unreliable criterion, but relief of pain is a prima facie evidence for making the diagnosis [22] The diagnostic injection permits testing of the hypothesis that the target structure (facet joint) is the source of a patient’s pain. Revel et al. [67] found that clinical signs are unsuitable for making a diagnosis, but may be of value in selecting patients for diagnostic block of the facet joint. Hence, the interventional/musculoskeletal radiologist and rheumatologist/spine specialist will need to work in close consultation to select the appropriate candidate for facet injection.

Indications may be classified as: (a) diagnostic and (b) therapeutic.

### Diagnostic

1. The primary indication is to confirm a clinical suspicion of the facet syndrome. Clinical signs include local paraspinal tenderness; pain that is brought about or increased on hyperextension, rotation, and lateral bending; absence of neurologic deficit; absence of root tension signs; and hip, buttock, or back pain when the straight leg is raised [53]. Symptoms of facet syndrome also include cramping leg pain involving the thigh but not radiating below the knee, low back stiffness, and absence of paraesthesia [53]. The back stiffness is typically most marked in the morning [68]. Low back pain is brought about or increased by maintenance of certain positions, such as sitting erect for a long period of time [69]. Focal tenderness over a facet joint is a strong indication in the appropriate settings, besides the presence of signs of paravertebral spasm or deformity in patients, with abnormal facet joints on imaging studies [61]. Cervical facet pain is often characterised by chronic headaches, restricted motion and axial neck pain, which may radiate sub-occipitally to the shoulders or mid-back [70].
2. Multilevel spinal involvement in a chronic pain process after excluding other obvious causes, conflict between the location of imaging abnormalities and clinical symptoms, and for pre-surgical testing [66].
3. Chronic low back pain (lumbar facets) and chronic neck pain (cervical facets) that are not relieved by trial of routine non-steroidal anti-inflammatory drugs (NSAIDs) and physiotherapy.
4. Post-laminectomy syndrome with no evidence of arachnoiditis or recurrent disc disease [18].
5. Persistent low back pain after a stable posterolateral fusion [63].
6. Post-whiplash injury, chronic neck pain [70].
7. Low back pain, with or without sciatica, but with normal radiological findings [63, 69].

### Therapeutic

1. Patients with confirmed facet syndrome especially those who respond to diagnostic facet injection.
2. As an adjunct to conservative management.
3. In patients on oral or systemic drug therapy if these drugs have to be discontinued because of their adverse effects or if they have reached their maximum tolerable dose.
4. Presence of adjacent segment deterioration after spinal fusion [66].
5. Pain due to attrition across spondylolytic defects [18, 69].

## CONTRAINDICATIONS [55, 56]

Exclusion criteria are relative with no absolute contraindications. Injections are generally avoided in patients with: systemic infection or skin infection over puncture site; bleeding disorders or coagulopathy; allergy to contrast agents or any of the medications to be administered; inability to obtain percutaneous access to the target facet joint; progressive neurological disorder which may be masked by the procedure; and pregnancy.

## TECHNIQUE, DRUGS AND EQUIPMENT

The procedure is usually done on an outpatient basis. Before the start of the procedure, the patient should be interviewed about the type, location and nature of the pain, and any history of prior surgery. Pain drawings may be helpful in identifying the specific levels that are associated with the patient’s complaints. The patient’s medical and imaging records should be carefully reviewed, and the magnetic resonance (MR) images should be compared with radiographs to evaluate for possible level ambiguity due to a transitional lumbosacral segment. When obtaining an informed consent, ensure that the patient understands the purpose of the procedure, the risks involved and most importantly, are enlightened about the variable therapeutic outcomes. The patient needs to be aware that unlike most of the other therapeutic interventions, the outcome of this procedure is highly variable and they may not receive the desired benefit. Similarly, they must be aware of the transient nature of the therapeutic benefits and there may be a need for repeated injections. In the author’s practice, the patient comes fasting for 6 to 8 hours prior to the procedure. Although premedication is not routinely used, a mild sedative may be administered in anxious patients. Sedation is avoided as far as possible, since it may interfere with the patient’s response to pain reproduction. The procedure is performed with strict aseptic precautions and intra-procedural regular monitoring of the vital parameters is recommended. Facet joint injection is best performed in an interventional suite within the diagnostic radiology department. High-quality C-arm fluoroscopy is preferred. The type of image guidance used for facet injection is a matter of personal preference and depends on the expertise of the operator. In the personal experience of the author, the procedure time is shorter for fluoroscopy-guided injections and radiation exposure is also lower. Alternatively, ultrasound, CT and MR guidance may also be used for the needle placement.

The facet joint injection set that is used in the author’s institute consists of: a 23G spinal needle for entering the facet joint; a 18G needle for aspirating drugs; a 24G hypodermic needle for skin infiltration with lignocaine; a 1ml insulin syringe for delivering the injectate into the joint; 40mg% Triamcinolone (a long-acting steroid depot preparation) and 0.5% Bupivacaine (long-acting local anaesthetic agent). The local anaesthetic and steroid were mixed in equal volumes. The local anaesthetic used here should be preservative-free, so as to prevent flocculation of the steroid. Other steroids like methyprednisolone or betametasone may be employed. 1% Lignocaine is used for skin infiltration, and 300 mg% Iohexol as the contrast agent. In the majority of cases, anatomical location of the needle tip on fluoroscopic imaging is sufficient for localisation. Contrast arthrography is not performed in all the patients since the author’s practice does not insist on precise intra-articular injection. From anecdotal experience, periarticular injections give equivalent results as endorsed by certain published studies [39, 71, 72]. In thick patients, a longer spinal needle may be required. In some instances, even co-axial insertion of the spinal needle through an 18 or 20 G stiff metallic needle (with stillete) may be required, as manipulation of the spinal needle through a thick layer of muscle and fat may not be always possible.

The biggest problem is the initial clinical determination of those patients who may have facet syndrome and the decision as to which level(s) to inject. Hence, multiple injections are often required. The local anaesthetic agent within the injectate may act on the nociceptive fibres in the synovium, whereas intracapsular corticosteroids may reduce inflammation of the synovium [63]. In such targeted injections, the anaesthetic is probably responsible for immediate pain relief, whereas steroids are believed to be responsible for pain relief 2–6 days after their administration [73]. For a diagnostic block, a short-acting anaesthetic alone may be sufficient. A long-acting agent like bupivacaine is preferred for therapeutic injections since it has an added advantage of prolonging the nerve conduction blockage of the nociceptive fibres, thereby breaking the pain cycle for longer durations [54]. Presumably, if this interruption of the pain cycle is of adequate duration, the natural mechanisms may overcome or compensate for the cause of pain, and the patient may yield better therapeutic results. The role of steroids is controversial, with some studies showing no advantage from the addition of steroids to the injectate [74]. However, it is advisable to follow the recommendation on the upper limit of steroid usage (3mg/kg of body weight of steroid, or 210mg per year in an average person and a lifetime dose of 420 mg of steroid, equivalent to methylprednisolone) [75]. In the diagnostic phase, a patient may receive injections at intervals of no less than one, week, though a two–week interval is preferable. In the therapeutic phase, the suggested interval between injections is two months, provided that at least > 50% relief is obtained for 6 weeks [75].

## LUMBAR INTRA-ARTICULAR FACET INJECTIONS (FIGURES 2, 3, 4)

Patient lies prone and centering is done at the spinal level of interest.

**Figure 4:**
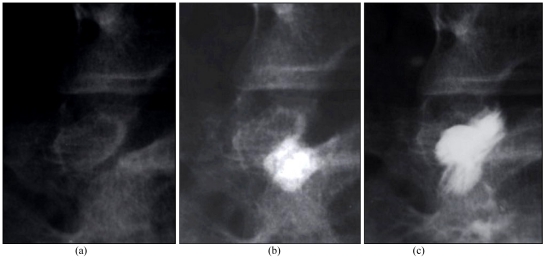
Left-sided L3/L4 facet joint is seen in profile (arrows). Also note the needle seen end-on within the ipsilateral L4/L5 facet joint.

The patient is made to turn gently so that the side to be injected is raised off the couch, till the joint is seen in profile (Figure 4). This position is now secured. Alternatively, the tube may be tilted to obtain an equivalent projection of the facet.

The facet joints are curved structures. The superior facets face anterolaterally while the inferior facets face posteromedially. Hence, the posterior aspect of the joint lies further from the midline than the anterior aspect [42, 76]. During postero-anterior fluoroscopy, as we gently rotate the patient through an arc, the first portion of the joint seen is its posterior aspect. Over-rotation may bring the anterior portion of the joint in profile. This has to be avoided since seeing the anterior joint margin in profile will make needle placement into the joint impossible. The upper lumbar spine may require obliquity of as little as 30 degrees, while the lower lumbar spine may require obliquity of up to 60 degrees (Figure 3). Once localised, the needle may be to be directed vertically into the centre of the facet joint space.

Alternatively, one may also use the technique described by Sarazin et al. [61]. They perform the procedure with the patient prone, and the needle is advanced towards the lower pole of the inferior apophyseal process, as this is the expected location of the inferior recess of the facet joint. The inferior recess is preferentially targeted since it is located posteriorly, has no direct relation with neural elements, is relatively capacious and is easy to enter. This approach is more useful in the presence of obstructing osteophytes. In a kyphotic posture, this space widens, hence pillows may be placed below the abdomen to generate kyphosis. If the spine is osteoporotic and the inferior apophyseal process is not seen well, the target point is located at the medial projection of the vertebral pedicle.

Once the site for entry is selected, the skin is marked at this position and local anaesthetic infiltrated into the skin and superficial muscles. The spinal needle is then inserted vertically through this point until it reaches the bone. Once it enters the joint, a “giving way” sensation is perceived. Some minor manipulation may be needed to get into the joint space.

The intra-articular location of the needle tip is be confirmed by rotating the patient and observing the needle tip move together with the facet joint. Alternatively, a facet arthrogram may be done. If the needle tip is intra-articular in position, the contrast flows away from the needle tip during injection [69], extending into the superior aspect of the facet joint or opacifying one of the recesses located in a dependent part of the joint capsule. Usually no more than 0.2 ml of contrast is injected, as it can restrict the amount of drug mixture that has to be injected later. The joint capsule is smooth and oval in the frontal projection and S-shaped in the oblique projection. If the contrast agent accumulates as a blob, the needle is likely to be at the wrong site; do not inject more as it can smudge the field. In the lumbar spine, the arthrogram can often show variable pictures, with the contrast agent sometimes going into the contralateral facet and sometimes into the facet joint above. If there is spondylolysis, the defect in the part interarticularis may be opacified [77]. A positive block is useful in the diagnosis of pars defect-related pain, particularly when spinal fusion surgery is being considered.

Once the position of the needle tip is confirmed, a combination consisting of 1 to 1.5 ml of the steroid and local anaesthetic, in equal parts, is injected. Always aspirate before injection, to exclude accidental intravascular location of needle. The volume of the joint usually does not exceed 1.5 to 2ml. We usually terminate the injection when resistance is encountered. Large volume injections may cause rupture of the joint capsule and extravasation of the injectate into the epidural space; this may account for part of the therapeutic effect described in certain studies [78].

Periarticular injection is an acceptable alternative, should reasonable attempts to gain an intra-articular position prove difficult. During a periarticular injection, the needle is rotated 360 degrees at the desired location and 1.0–1.5ml of the steroid and local anaesthetic mixture is injected. In the author’s experience, the outcome is fairly the same; meanwhile, in published literature, there is disagreement as to whether intraarticular injection is preferable to periarticular injection [39, 79–81].

## THORACIC INTRA-ARTICULAR FACET INJECTIONS

The orientation of the facet joints changes, descending along the spinal column from a coronal angulation in the cervical spine to a sagitally oblique orientation in the lower lumbar spine. At the thoracolumbar junction, there is a gradual change in the orientation of the joints, from coronal to sagittal. Thoracic facet joints are nearly vertical and coronal in orientation [44]. They are inclined nearly 60 degrees to the coronal plane and rotated so that the superior articular facet faces posteriorly, superiorly and laterally [76]. Hence the lateral aspect of the joint is located anteriorly, whereas the medial side of the joint is located more posteriorly. With their sagittal orientation, the lower thoracic facet joints more closely resemble the lumbar facets.

In the thoracic spine, the basic technique and approach of facet joint injection is similar to that of the lumbar spine, with adequate concession to the differences in their anatomical orientation. As the medial aspect of the joint is more superficial and posterior, the needle is targeted towards it. With the patient prone, the point of skin entry is marked at the middle of the pedicle of the vertebra one level below the facet being injected (for example, if T6-T7 facet has to be injected, the skin entry site is at the middle of the pedicle of T8). Puncture the skin at a 60-degree angle and advance the needle cranially towards the target joint. The track of the needle is maintained between the imaginary planes along the lateral and medial border of the pedicle. This is to avoid accidental puncture of the thecal sac or lungs. Along this course, once the needle tip reaches the upper border of the pedicle, one vertebral level above the point of skin entry (if we consider the aforementioned example, it would beT7 pedicle) rotate the C-arm (almost lateral) to visualise the joint in profile. If the track is appropriate, the needle tip should be seen just below the posteroinferior aspect of the target joint that may be subsequently entered. In the thoracic spine it is recommended to inject a drop of contrast and confirm the intra-articular location of the needle, since the overlapping bony structures and steep angle of entry makes assessment of its location difficult in plain fluoroscopy.

Compared to cervical or lumbar facet injection, thoracic facet joint injection is technically more challenging; hence CT is preferred in difficult cases or in patients with extensive degenerative changes and spinal deformities.

## CERVICAL INTRA-ARTICULAR FACET INJECTIONS

The facet joints in the cervical spine are oriented in an oblique coronal plane, with 35–45 degrees of posterior slope [44, 76]; hence, a lateral approach is preferred. Some interventionists use an oblique frontal approach. The patient is placed in a lateral position with the side to be injected facing upwards, shoulders drawn down and placed on a head rest to avoid lateral flexion of the neck. Once the joint is viewed in profile, it is entered from the posterior and inferior aspect under direct vision. During cervical facet injections it is most critical to avoid inadvertent entry into major vessels, a nerve root sheath or subarachnoid space. Extra care should be taken to ensure that the needle tip is directly positioned over the posterior elements on the lateral projection, avoiding in particular the inter-vertebral foramina and spinal canal. For the less experienced practitioners, frequent intermittent fluoroscopy in the frontal projection is important to check the depth of the needle.

The same injectate is used at all the levels. However, the thoracic and cervical facet joints are presumably less capacious, as an increase in resistance to injection has been observed after delivering a smaller volume of the drug than at the lumbar facet. This is also recorded in published literature (56). To avoid the risk of prolonged effects on vital cervicomedully centres in case of an accidental subarachnoid injection, some operators preferentially use lignocaine instead of bupivacaine for the cervical facets.

## POST-PROCEDURE CARE AND COMPLICATIONS

Post-procedurally, patients are routinely monitored for 15–20 minutes and then discharged home. The response to injection is recorded soon after infiltration. The response is recorded in the same format as in pre-injection, using a visual analogue scale (VAS) that enables better quantification of the response to treatment.

Complications are rare, particularly if the facet joint injection is performed using a precise needle-positioning technique [64, 82, 83]. Possible complications include spondylodiscitis [84], septic arthritis [85], and reaction to the injectates. Septic arthritis can be avoided with appropriate aseptic precautions. Severe allergic reactions to local anaesthetics are uncommon. Steroid injection may produce local reactions, occurring most often immediately after injection. These local reactions last for 24–48 hours, and are relieved by application of ice packs [69]. Post-procedural pain flare-up may occasionally occur, and may be treated with NSAIDS. Transient numbness and paralysis, as well as paraesthesia, are expected to resolve within minutes to hours. Event transient tetraplegia after cervical facet injection has also been reported [86]. Other possible complications include vertebral artery damage during cervical entry, leakage of anaesthetic into the spinal canal causing motor and sensory blockade, phrenic palsy from overflow of local anaesthetic during injection at C3-C6 levels [75], chemical meningitis [87], epidural abscess [88], pneumothorax, haematoma formation, as well as transient ataxia and unsteadiness due to partial blockade of upper cervical proprioceptive afferents and the righting reflex from the third occipital nerve block during cervical injections [89, 90]. Significant vascular and neurological injuries are, however, extremely rare in image-guided injections.

## CLINICAL OUTCOME

When performed under fluoroscopic visualisation, facet joint injections are accurate and clinically useful in the diagnosis and therapeutic management of chronic spinal pain. The diagnostic accuracy of facet joint blocks is strong for cervical and lumbar facet joints, and moderate for thoracic facet joints [64]. In contrast to clinical evaluation and imaging techniques, diagnostic injections can identify facet joint pain with a remarkably higher level of certainty [91]. However, the diagnostic value is limited by the high false-positive rates seen with single blocks (without control). False-positive rates with single blocks are 17%- 47% in the lumbar spine, 27%-63% in the cervical spine, and 55%-58% in the thoracic spine [67, 75, 91]. There is limited literature on the therapeutic efficacy as most of the available data is based on non-controlled and observational studies. There are only few exhaustive randomised control trials available on this subject and they mostly pertain to the lumbar spine. Controlled studies for lumbar facet injections have shown initial relief of symptoms (1–4 weeks) in 42 to 92 % of patients [81, 92], while long-term relief at three months varies between 18% and 62% [81, 93]; meanwhile uncontrolled observational studies have shown short-term response up to 94% [62]. In conclusion, for intra-articular injection of local anaesthetics and steroids, there is moderate evidence of short-term relief and limited evidence of long-term relief of chronic neck and low back pain [41]; meanwhile a recent systematic review failed to provide any evidence or recommendation with respect to the therapeutic value of intra-articular facet joint injections in the thoracic spine [94].

The evidence of intra-articular injections of local anaesthetics and steroids from randomised trials, complemented with that of non-randomised trials (prospective and retrospective evaluations), provided moderate evidence of short-term relief and limited evidence of long-term relief of chronic neck and low back pain.
